# Integrated physiological, proteomic, and metabolomic analyses of pecan cultivar ‘Pawnee’ adaptation to salt stress

**DOI:** 10.1038/s41598-022-05866-9

**Published:** 2022-02-03

**Authors:** Yun Jiao, Jianhong Zhang, Cunde Pan

**Affiliations:** 1 Institute of Forestry, Ningbo Academy of Agricultural Science, No. 19 Dehou Street, Ningbo, 315040 Zhejiang People’s Republic of China; 2 College of Forestry and Horticulture, Xinjiang Agricultural University, No. 311 Nongda East Road, Ürümqi, 830052 Xingjiang People’s Republic of China

**Keywords:** Physiology, Proteomics, Salt, Secondary metabolism

## Abstract

The pecan is a salt-alkali-tolerant plant, and its fruit and wood have high economic value. This study aimed to explore the molecular mechanisms responsible for salt stress tolerance in the pecan grown under hydroponic conditions to simulate salt stress. The results showed that the photosynthetic rate (*P*n) was reduced in response to salt stress, while the intercellular carbon dioxide concentrations (*C*i) increased. The response of the pecan to salt stress was measured using iTRAQ (isobaric tags for relative or absolute quantitation) and LC/MS (liquid chromatography and mass spectrometry) non-targeted metabolomics technology. A total of 198 differentially expressed proteins (65 down-regulated and 133 up-regulated) and 538 differentially expressed metabolites (283 down-regulated and 255 up-regulated) were identified after exposure to salt stress for 48 h. These genes were associated with 21 core pathways, shown by Kyoto Encyclopedia of Genes and Genomes annotation and enrichment, including the metabolic pathways involved in nucleotide sugar and amino sugar metabolism, amino acid biosynthesis, starch and sucrose metabolism, and phenylpropane biosynthesis. In addition, analysis of interactions between the differentially expressed proteins and metabolites showed that two key nodes of the salt stress regulatory network, L-fucose and succinate, were up-regulated and down-regulated, respectively, suggesting that these metabolites may be significant for adaptations to salt stress. Finally, several key proteins were further verified by parallel reaction monitoring. In conclusion, this study used physiological, proteomic, and metabolomic methods to provide an important preliminary foundation for improving the salt tolerance of pecans.

## Introduction

Leaves are the primary sites of photosynthesis and energy exchange in plants, and are highly sensitive to environmental conditions^1,2^. Salt stress can affect plant growth by reducing photosynthesis, with more severe effects seen with higher salt concentrations and longer exposure times directly or indirectly, resulting in reduced photosynthesis^3^. Transcriptomic analysis of pecan leaves in the early stages of salt stress has identified a large number of genes that respond to salt stress^4^. However, simple transcriptomic analysis cannot provide a comprehensive analysis of the molecular mechanism of salt stress adaptation, which requires in-depth investigation based on proteomics and metabolomics.

Quantitative proteomic methods, such as isobaric tag for relative and absolute quantitation (iTRAQ) and metabolomic techniques, such as liquid chromatography and mass spectrometry (LC/MS) allow in-depth analyses of plant metabolic adaptations to salt stress. These platforms have been used to integrate and analyze changes in barley (*Hordeum spontaneum* L.)^5^, *Arabidopsis thaliana*^6^, wheat^7^, *Malus halliana*^8^, rice^9^, maize^10^, small rust fungus^11^, and sugar beet^12^ under salt stress conditions. For instance, significant changes in the contents of soluble sugars were found after wheat seedlings were inoculated with the wp-6 strain (plant growth-promoting rhizosphere bacteria) under salt stress; these included proteins related to energy production and conversion and the metabolites palmitoleic acid, chlorophyll b, D-arginine, pheophytin a, rutin, and vanillin, suggesting that these may play important roles in promoting the growth of salt-stressed wheat seedlings^7^. Similarly, the salt-tolerant sugar beet T510 variety showed increased growth, together with higher sucrose and proline levels, and increased antioxidant enzyme activity in response to salt stress. The differentially expressed proteins (DEPs) identified by iTRAQ in sugar beet were found to be involved in many different activities, including photosynthesis, stress and defense, protein synthesis, and signal transduction^12^. It is apparent that plants accumulate sugar, proline, secondary metabolites, and solutes related to osmotic adjustment on exposure to salt stress, as well as developing adaptive strategies and increasing their antioxidant capabilities, which the intrinsic mechanism of plant in response to salt stress has been recognized by many researchers. Interestingly, sugar not only participates in osmotic regulation, but also acts as a signaling molecule, sensing environmental stress and activating defense responses through the regulation of gene and protein expression and metabolite accumulation. For instance, sucrose promotes the accumulation of tryptophan, certain alkaloids, and D-phenylalanine, and can also activate glutamate-related proteins (ASN1, ASP3, GLN1-1, and NIT4) and aspartic acid, leading to the control of reactive oxygen species (ROS) levels and the induction of flavonoid biosynthesis and auxin signaling^8^. These reactions enhance the resistance of *M. halliana* to salt-alkali stress. Furthermore, glucose and fructose, acting as signaling molecules, have been shown to activate downstream salt-stress responses by regulating the expression of specific proteins^13,14^.

In addition, some proteins, such as heat shock proteins (HSPs)^15^, mitogen-activated protein kinases (MAPKs)^16^, and the photosynthetic protein PSBO2^17^, are thought to be involved in a variety of signal transduction pathways related to the salt-stress response, thereby increasing resistance to abiotic stress. For instance, MAPKs play crucial roles in the regulation of transcription factor gene expression under salt stress in rice^18^. Hsp17.6CII (a chaperone protein) in *Arabidopsis* activates CAT2 activity by regulating ROS levels, thus increasing the plant’s resistance to abiotic stress^19^. Furthermore, several small molecules, including melatonin^20^ and γ-Aminobutyric acid (GABA)^21^, can enhance resistance to salt stress by triggering the expression of downstream proteins involved in the salt-stress response. Thus, the ability of halophytes to tolerate high salt levels is determined by a coordinated network of proteins and metabolites.

Current research on the molecular mechanisms of salt stress has concentrated mostly on model plants, and there are few studies on perennial woody oil plants. In this study, hydroponics was used to simulate the salt-stress environment to comprehensively analyze the effects of salt stress on pecans. The physiological parameters relating to photosynthesis in the pecan leaves were analyzed, and used iTRAQ proteomics and non-targeted LC/MS metabolomics to investigate the molecular regulatory mechanisms of pecans in response to salt stress with the goal of providing a theoretical basis for the future establishment of pecan cultivation in saline-alkali soils.

## Experimental details

### Plant material, salt-stress treatment, and analysis of photosynthetic parameters

Pecans were sourced and processed as previously described^4^. Specifically, samples of fully expanded leaves were collected from the plants and were then divided into three groups, with each group containing three replicates of six leaves. The groups were subjected to different NaCl concentrations and incubation times, as follows: group A, control group, no NaCl treatment, 0 h; group B, 0.6% NaCl for 24 h; group C, 0.6% NaCl for 48 h. The samples were then rapidly frozen in liquid nitrogen for subsequent analysis. These analyses included the measurement of the photosynthetic rate (*P*n), stomatal conductance (*G*s), and intercellular CO_2_ concentration (*C*i), measured on an LI-6400XT portable photosynthetic rate meter (Li-COR Biosciences, Lincoln, USA), on functional leaves at 10:00–11:00 (under natural light) on six leaves with three replicates per group (as below). At the same time, maximal fluorescence (Fm), the initial fluorescence (F0), maximal photochemical efficiency (Fv/Fm), light-induced nonphotochemical quenching (NPQ), nonregulatory energy dissipation (Y(NO)), and photochemical quenching coefficient (qN) were measured in the leaves after incubation for 30 min in the dark using a chlorophyll fluorometer (PAM2500, Heinz Walz GmbH, Germany) and Pam-Win3 software. These values were exported to Excel (Microsoft Excel 2016) and analyzed using one-way ANOVA with *p* values < 0.05 considered statistically significant, using the Data Processing System software (version 14.10, Zhejiang University, Hangzhou, China). Finally, Adobe Illustrator 2019 (Adobe Systems, San Jose, CA, USA) and OriginPro software version 2021 (Northampton, MA, USA) were used to draw and edit the data maps.

### Isobaric tags for relative and absolute quantitation (iTRAQ) analysis

Protein extracts from three biological replicates from each treatment group were used for digestion and iTRAQ labeling, as previously described^12^. The protein concentration was determined by the bicinchoninic acid (BCA) method^22^. The iTRAQ labeling was carried out according to the filter aided sample preparation (FASP) method^23^, and the components separated using an Agilent 1100 HPLC (Agilent Technologies, Palo Alto, CA, USA). The relevant parameters were as follows: flow rate, 300 μl/min; mobile phase A phase, H_2_O-FA (99.9:0.1, v /v); mobile phase B phase, ACN-H_2_O-FA (80:19.9:0.1, v/v/v); gradient elution conditions: 0–1 min, 2–9% B; 1–45 min, 9–29% B; 45–52 min, 29–37% B; 52–56 min, 37–100% B; 56–60 min, 100% B. The mass spectrometry conditions were described in the reference^24^. Finally, Proteome Discoverer 2.4 software (Thermo Fisher Scientific, Bremen, Germany) was used to complete the protein identification and analysis. The mass spectrometry proteomics data have been uploaded to the Proteome Xchange Consortium (http://proteomecentral.proteomexchange.org) with the dataset identifier PXD030031 and the subject ID IPX0003700000.

### LC/MS untargeted metabolomics analysis

Eight milligrams from each sample were weighed out, and 20 μl internal standard^25^, and 1 ml methanol: water (V:V = 7:3) were added, and ground in a grinder (60 Hz, 2 min). Ultrasonic extraction was performed for 30 min, after which the sample was allowed to stand at − 20 °C for 20 min and was then centrifuged for 10 min (13,000 rpm, 4 °C). Three hundred microliters of the supernatant were then evaporated to dryness, reconstituted with 400 μl methanol–water (V:V = 1:4), ultrasonicated for 2 min, and centrifuged for 10 min (13,000 rpm, 4 °C), before withdrawing 150 μl of the supernatant with a syringe. This was then filtered through a 0.22 μm organic phase pinhole filter. LC/MS analysis was performed using a liquid mass spectrometry system composed of Dionex U3000 UHPLC and QE plus (Thermo Fisher, Waltham, MA, USA). Data collection and the identification of compounds were described in the reference^26^. Metabolic changes in the samples were investigated using principal component analysis (PCA) and (orthogonal) partial least-square discriminant analysis (O) PLS-DA. The variable importance in projection (VIP) was used to rank the overall contribution of each variable to the OPLS-DA model, and those variables with *p* values < 0.05 and VIP > 1 were considered to be related to group discrimination. In addition, the Kyoto Encyclopedia of Genes and Genomes (KEGG, http://www.genome.jp/KEGG/pathway.html) was searched for metabolic pathway enrichment.

### Data analysis and verification of iTRAQ data by PRM

To analyze the functional characteristics of the selected DEPs, annotation information was extracted from the gene ontology (GO) and KEGG databases using the search term *Juglans regia* “English walnut”^27^for protein functional annotation and pathway enrichment analysis. In addition, the online analysis platform Paintomics 3 (http://www.paintomics.org/) was used for the correlation analysis between DEPs and metabolites, as well as the OmicShare online analysis cloud platform 6.4.5 (https://www.omicshare.com/tools/) to establish a network diagram of DEPs and metabolites. Among them, the core DEPs and metabolites were determined through the MetaboAnalyst 5.0 online analysis platform (https://www.metaboanalyst.ca/MetaboAnalyst/) to establish a regulatory network diagram. Furthermore, 20 proteins were chosen and quantified to verify the iTRAQ data by PRM analysis, with the detailed parameters for the liquid chromatography (LC) system and mass spectrometer taken from the relevant literature^28^. Three biological replicates of each group were performed. The PRM analysis is supported by Novogene Bioinformatics Institute (Beijing, China).

### Ethics approval

The experimental research and field studies on plants, including the collection of plant material, complied with relevant institutional, national, and international guidelines and legislation. The appropriate permissions and/or licenses for the collection of plant or seed specimens were obtained for the study.

## Results

### Pecan leaves in response to salt stress and changes in photosynthesis

**Figure 1:**
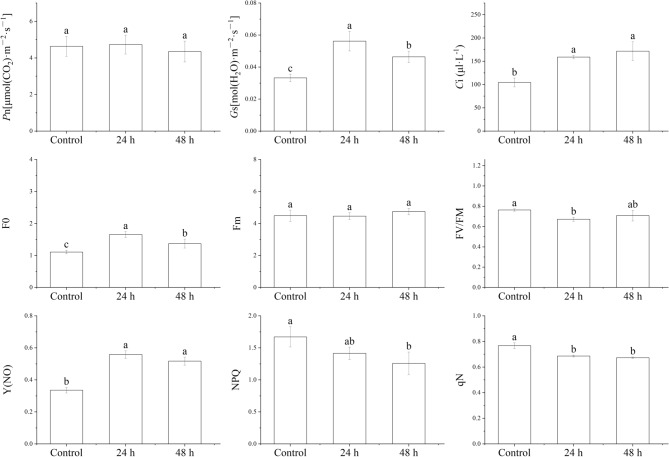
Physiological parameters of pecan under salt stress: photosynthetic rate (*P*n); stomatal conductance (*G*s); intercellular CO_2_ concentration (*C*i); initial fluorescence (F0); maximal fluorescence (Fm); maximal photochemical efficiency (Fv/Fm); light-induced nonphotochemical quenching (NPQ); nonregulatory energy dissipation (Y(NO)); photochemical quenching coefficient (qN).

To understand the physiological response of pecans to salt stress, we treated pecans with NaCl. After 24 h, the morphological characteristics of the pecan leaves were not significantly different from those of the control (CK). Only after 48 h could a slight lightening of the color on the leaf edges be seen (Fig. S1). In addition, the photosynthetic rate (*P*n) showed a downward trend after 24 h treatment but the overall change was not large (Fig. 1). The stomatal conductance (*G*s) showed an obvious upward trend after 24 h of salt-stress treatment. The intercellular carbon dioxide concentration (*C*i) of the leaves rose continuously with prolonged exposure to salt stress, implying that pecans may be mainly affected by stomal regulation. At the same time, the F0 and Y(NO) of the leaf fluorescence parameters showed an upward trend, indicating that the leaf tissue did not completely utilize the light energy absorbed by the plant, and that salt stress had already caused damage to the photosynthetic system. The decrease in Fv/Fm suggested a gradual increase in the photoinhibition response while NPQ and qN showed significant decreases after treatment for 24 h, indicating a gradual reduction in the photoprotective ability of the leaf tissue and an inability to convert excess light energy into heat loss. Thus, although the leaves did not show marked reactions to salt stress treatment, photosynthesis was inhibited to a certain extent.

### Proteomic analyses of pecan leaves in response to salt stress

**Figure 2:**
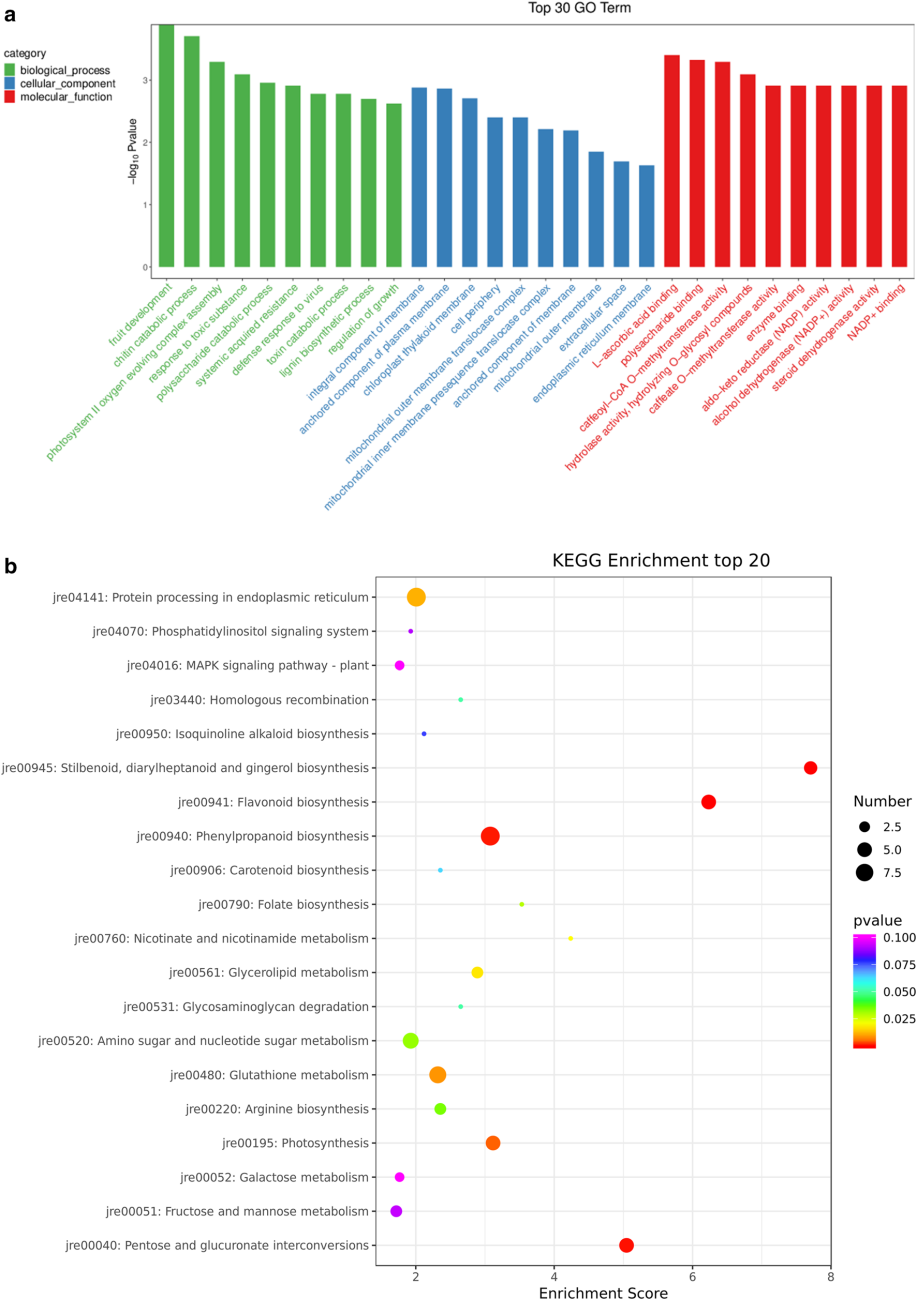
**A** GO classification of differentially expressed proteins under salt stress. Three main GO categories are summarized: BP, CC, and MF. BP, biological process; CC, cellular component; MF, molecular function. **B** The top 20 enriched gene ontology (GO) terms in the biological process under 0.6% NaCl stress for 48 h. The rich factor indicates the ratio of the number of DEPs in the pathway to the total number of genes in the pathway, and the higher the Rich factor, the higher the degree of enrichment.

Pecan leaves were evaluated using iTRAQ quantitative proteomics, and the adjusted *p* values < 0.05 were used as thresholds to screen for significant changes in DEP abundance. We identified 198 DEPs in the leaves; of these, 65 were down-regulated and 133 were up-regulated (Table S1). GO term enrichment analysis showed that the 198 DEPs were divided into three categories, including biological processes (BP), cellular components (CC), and molecular functions (MF) (Fig. 2A). The most abundant terms included fruit development, chitin catabolism, and photosystem II oxygen-evolving complex components in the BP category. The CC category included significant enrichment of membrane components, anchoring components of the plasma membrane, and the chloroplast thylakoid membrane. For the MF category, L-ascorbic acid-binding and polysaccharide-binding were the most abundant categories. The top 20 KEGG pathways are shown in Fig. 2B. The DEPs were mainly involved in the metabolic pathways phenylpropane biosynthesis, protein processing in the endoplasmic reticulum, photosynthesis, MAPK signaling pathway, amino sugar and nucleotide sugar metabolism, glutathione metabolism, and pentose and glucose conversion of aldehyde esters. These results indicate that proteins involved in the synthesis of metabolites respond to salt stress by regulating various metabolic pathways.

### Metabolic analyses of pecan leaves in response to salt stress

**Figure 3:**
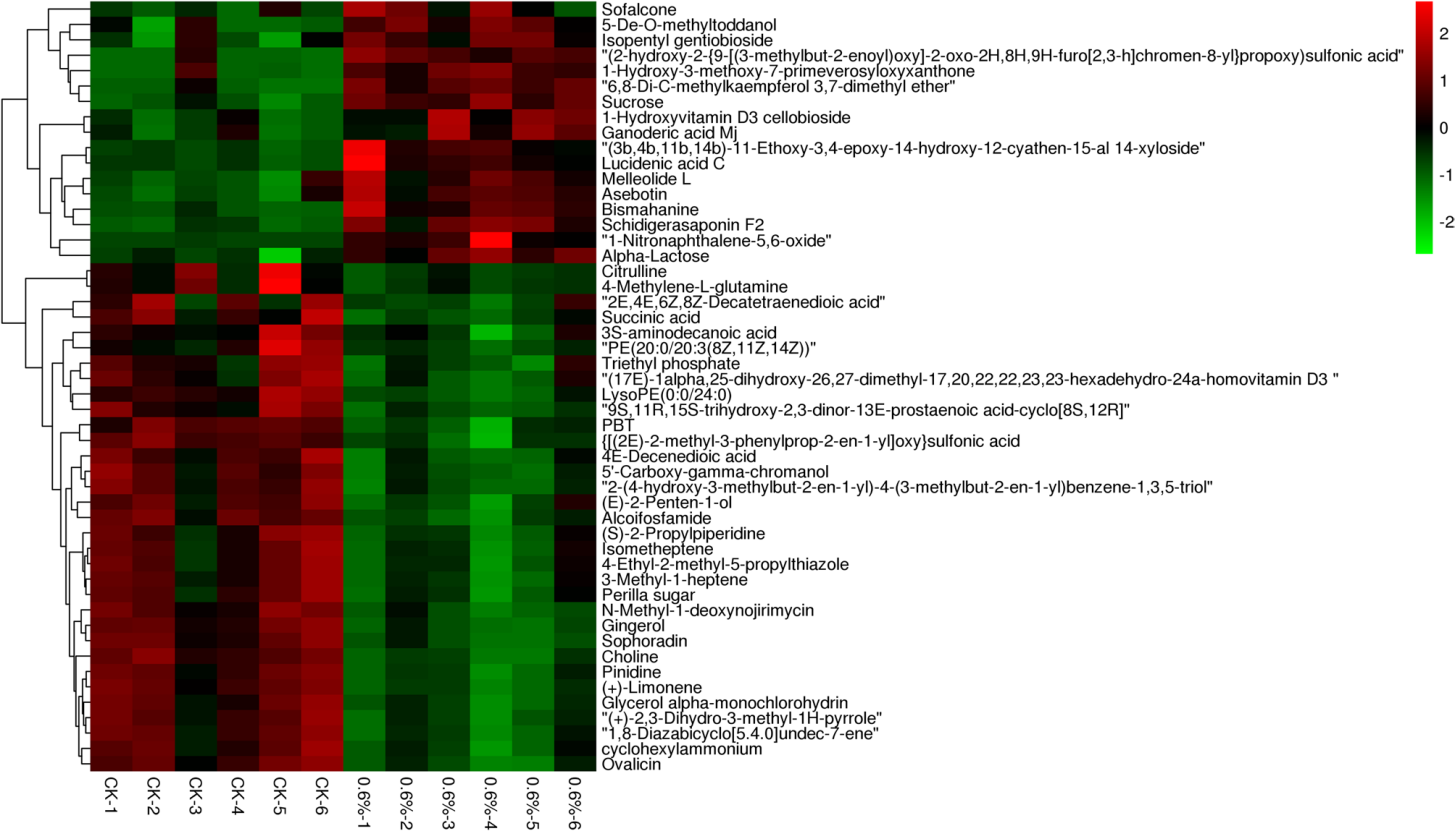
Hierarchical clustering heatmap of different metabolites in pecan leaves after 48 h of salt stress. The figure was carried out using the OmicShare online analysis cloud platform 6.4.5 (https://www.omicshare.com/tools/). "CK" stands for 0 h. “1–6” stands for repetition.

**Figure 4:**
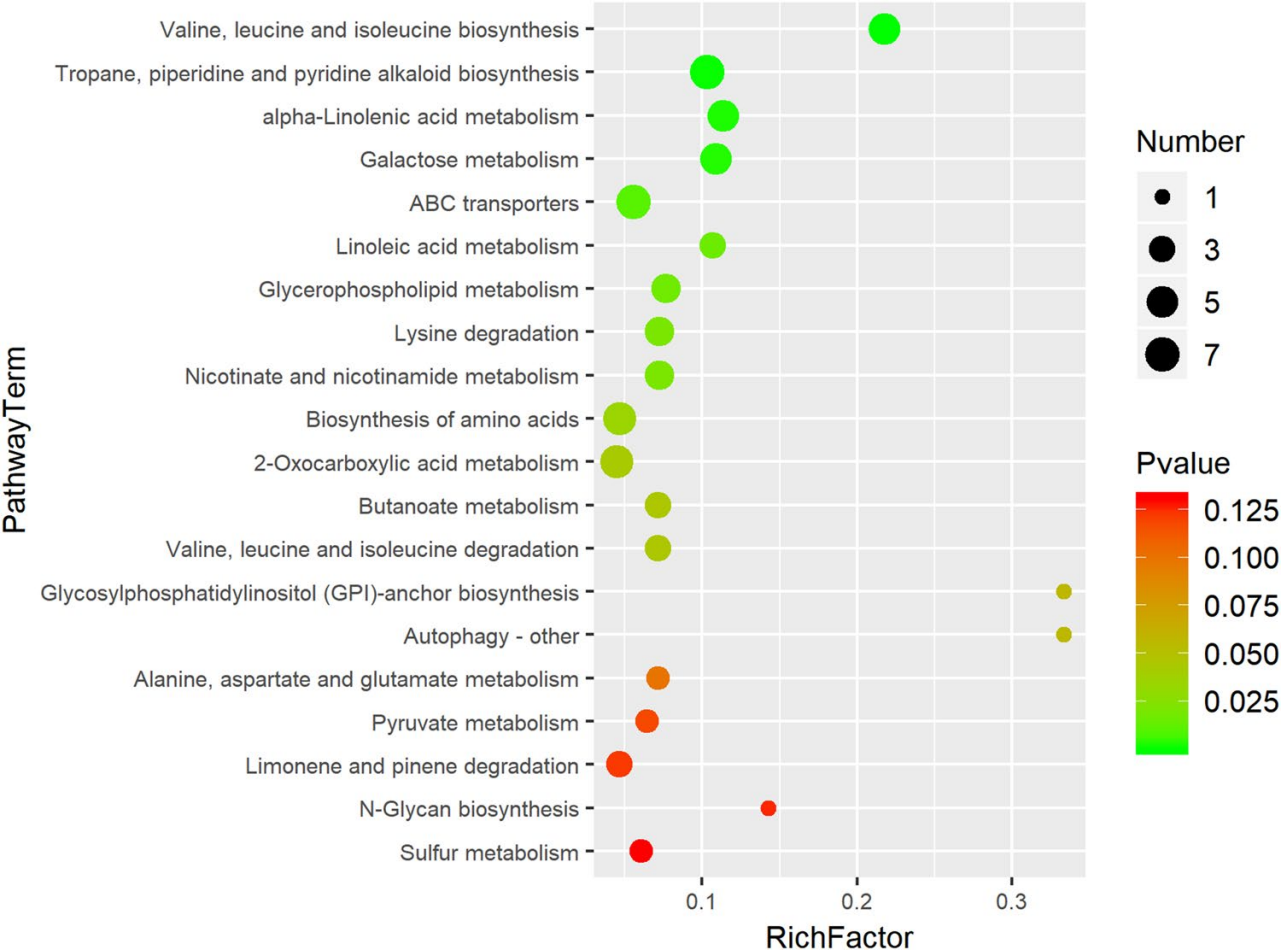
The top 20 enriched KEGG pathways under 0.6% NaCl stress for 48 h. The rich factor indicates the ratio of the number of DEMs in the pathway to the total number of DEMs in the pathway. A higher Rich factor indicates a greater degree of enrichment.

Through LC–MS identification and the OPLS-DA model with *p* value < 0.05 and VIP > 1, 538 differentially expressed metabolites (DEMs) were identified. Of these, 255 were up-regulated and 283 were down-regulated (Table S2). These metabolites mainly included amino acids, sugars, organic acids, and secondary metabolites. The top 50 DEMs were analyzed through hierarchical clustering; the heatmap is shown in Fig. 3. All the DEMs affected by salt stress were investigated in the KEGG database to identify the pathways in which they were involved. The top 20 pathways included piperidine and pyridine alkaloid biosynthesis, tropane, α-linolenic acid metabolism, galactose metabolism, ABC transporter, glycerophospholipid metabolism, lysine degradation, amino acid biosynthesis, 2-oxocarboxylic acid metabolism, and degradation of the limonene and pinene (Fig. 4). The changes in these metabolic pathways and metabolites provide important information to explain the response mechanism of pecans to salt stress.

### Correlation analysis of proteomics and metabolomics of leaves in response to salt stress

**Figure 5:**
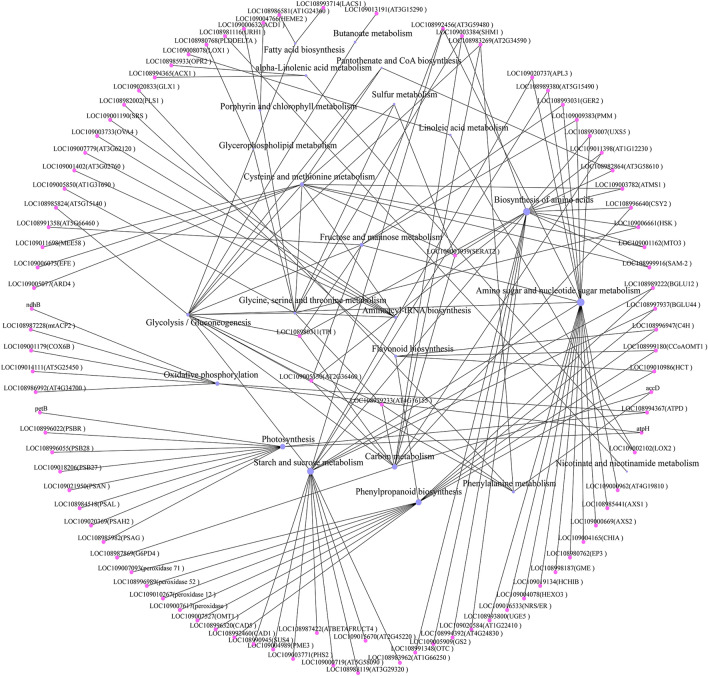
A summary of differentially expressed proteins related to the response of leaves to salt stress and the KEGG pathways involved. The protein is represented by a pink circle; the KEGG pathway it participates in is represented by a blue circle. The size of the blue circle represents the number of proteins involved in its pathway.

**Figure 6:**
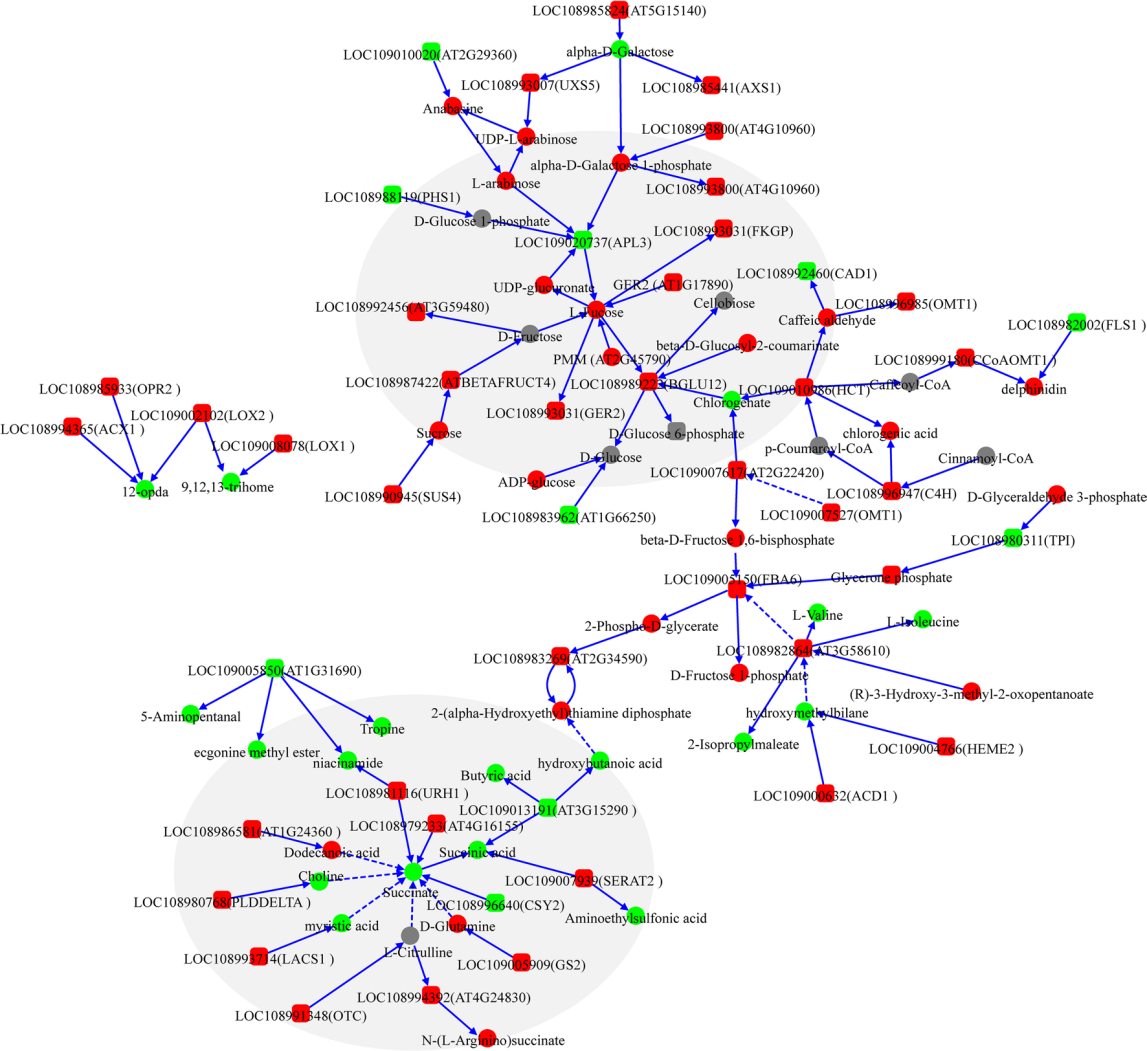
The core biological response pathway of pecan to salt stress. Based on the KEGG pathway, the DEM and DEP correlation analysis results are mapped to a comprehensive metabolic regulation network diagram. The DEMs and proteins are marked as circles and round rectangles, respectively; red indicates upregulation; green indicates downregulation. The key DEPs and DEMs are shown in the light gray background areas.

Analysis of the correlation between proteins and metabolites under salt stress showed that 23 metabolic pathways were enriched (Fig. 5); these included 93 proteins that were assigned to these pathways. These metabolic pathways included nucleotide sugar and amino sugar metabolism, starch and sucrose metabolism, amino acid biosynthesis, and phenylpropane biosynthesis. A regulatory network diagram of the core DEPs and DEMs included 21 metabolic pathways, indicating the response of the pecan leaves to salt stress (Fig. 6, Table 1). It can be clearly seen that there was a continuous accumulation of L-fucose, sucrose, L-arabinose, and beta-D-glucosyl-2-coumarinate in response to salt stress. Of these, L-arabinose (log_2_ Fold change = 3.96) and beta-D-glucosyl-2-coumarinate (log_2_Fold change = 2.70) showed significantly raised levels, and together with L-fucose, were the key nodes in the salt stress regulatory network. In contrast, succinate was down-regulated. Since the succinate participates in multiple metabolic regulation pathways and is a downstream component of metabolic regulation, indicating that metabolic pathways involving succinate may be key to the response to salt stress. In addition, the down-regulation of LOC109005850 (AT1G31690) expression involving the tropane, piperidine, and pyridine alkaloid biosynthetic pathway suggested that it could be involved in the reduced accumulation of four metabolites, namely, 5-aminopentanal, ecgonine methyl ester, niacinamide, and tropine. However, further verification is required.

**Table 1:** The core KEGG biological pathway and its changes in the correlation analysis results of DEP and DEM in pecan after 48 h of salt stress.

Pathway		Log_2_ (Fold change)
**Tropane, piperidine and pyridine alkaloid biosynthesis**		
Protein	LOC109005850/primary amine oxidase-like	− 0.25
	LOC109010020/tropinone reductase homolog	− 0.27
Metabolite	5-Aminopentanal	− 0.37
	Anabasine	1.96
	Ecgonine methyl ester	− 0.98
	Tropine	− 0.87
**Amino sugar and nucleotide sugar metabolism**		
Protein	LOC109020737/glucose-1-phosphate adenylyltransferase large subunit 1-like isoform X1	− 0.91
	LOC108993031/GDP-L-fucose synthase 1-like	0.44
	LOC108985441/UDP-D-xylose synthase 2-like	0.34
	LOC108993007/ UDP-XYL synthase 5	0.40
	LOC108993800/ UDP-D-glucose/UDP-D-galactose 4-epimerase 5	1.40
Metabolite	Alpha-D-Galactose 1-phosphate	2.44
	L-arabinose	3.96
	UDP-L-arabinose	0.74
**Starch and sucrose metabolism**		
Protein	LOC108983962/ O-Glycosyl hydrolases family 17 protein	− 0.46
	LOC108989222/beta-glucosidase 12-like	0.47
	LOC108990945/sucrose synthase-like	0.66
	LOC108988119/alpha-1,4 glucan phosphorylase L isozyme, chloroplastic/amyloplastic	− 0.28
	LOC108987422/acid beta-fructofuranosidase 1, vacuolar-like	0.29
	LOC109020737/glucose-1-phosphate adenylyltransferase large subunit 1-like isoform X1	− 0.91
Metabolite	Sucrose	2.84
**Phenylpropanoid biosynthesis**		
Protein	LOC108989222/beta-glucosidase 12-like	0.47
	LOC109010986/shikimate O-hydroxycinnamoyltransferase-like	0.34
	LOC109007617/peroxidase 17-like	0.27
	LOC108996985/caffeic acid 3-O-methyltransferase	0.39
	LOC108992460/probable cinnamyl alcohol dehydrogenase 1	− 0.54
	LOC109007527/caffeic acid 3-O-methyltransferase	0.49
Metabolite	Chlorogenate	− 1.95
	Beta-D-Glucosyl-2-coumarinate	2.70
	Cis-beta-D-Glucosyl-2-hydroxycinnamate	3.01
**Fructose and mannose metabolism**		
Protein	LOC108980311/ Triose phosphate isomerase	− 0.31
	LOC108993031/GDP-L-fucose synthase 1-like	0.44
	LOC109005150/fructose-bisphosphate aldolase 6, cytosolic	0.35
Metabolite	L-Fucose	2.74
**Stilbenoid, diarylheptanoid and gingerol biosynthesis**		
Protein	LOC108996947/trans-cinnamate 4-monooxygenase-like	0.34
	LOC108999180/caffeoyl-CoA O-methyltransferase 5	0.71
	LOC109010986/shikimate O-hydroxycinnamoyltransferase-like	0.34
Metabolite	Chlorogenic acid	1.19
**Arginine biosynthesis**		
Protein	LOC108991348/ornithine carbamoyltransferase, chloroplastic	0.26
	LOC108994392/argininosuccinate synthase, chloroplastic-like	0.30
	LOC109005909/glutamine synthetase leaf isozyme, chloroplastic-like	0.37
Metabolite	N-(L-Arginino)succinate	1.97
	D-Glutamine	0.85
**Galactose metabolism**		
Protein	LOC108993800/UDP-glucose 4-epimerase GEPI48	1.40
	LOC108987422/acid beta-fructofuranosidase 1, vacuolar-like	0.29
	LOC108985824/galactose mutarotase-like	0.37
Metabolite	Alpha-D-Galactose 1-phosphate	2.44
	Alpha-D-Galactose	0.65
**Citrate cycle (TCA cycle)**		
Protein	LOC108996640/citrate synthase, glyoxysomal-like	− 0.83
	LOC108983269/pyruvate dehydrogenase E1 component subunit beta-3, chloroplastic	0.28
	LOC108979233/dihydrolipoyl dehydrogenase 2, chloroplastic-like	1.21
Metabolite	Succinate	− 1.09
	2-(Alpha-Hydroxyethyl) thiamine diphosphate	2.14
**Glycolysis or gluconeogenesis**		
Protein	LOC108983269/pyruvate dehydrogenase E1 component subunit beta-3, chloroplastic	0.28
	LOC109005150/fructose-bisphosphate aldolase 6, cytosolic	0.35
Metabolite	2-Phospho-D-glycerate	1.88
**Valine, leucine, and isoleucine biosynthesis**		
Protein	LOC108982864/ketol-acid reductoisomerase, chloroplastic	0.41
Metabolite	(R)-3-Hydroxy-3-methyl-2-oxopentanoate	1.30
	L-Isoleucine	− 0.78
	L-Valine	− 3.04
	2-Isopropylmaleate	− 2.62
**Alpha-Linolenic acid metabolism**		
Protein	LOC108994365/peroxisomal acyl-coenzyme A oxidase 1	0.43
	LOC109002102/linoleate 13S-lipoxygenase 2–1, chloroplastic-like	0.34
	LOC108985933/12-oxophytodienoate reductase 2-like	0.41
Metabolite	12-Opda	− 0.96
**Linoleic acid metabolism**		
Protein	LOC109008078/probable linoleate 9S-lipoxygenase 5	0.53
	LOC109002102/linoleate 13S-lipoxygenase 2–1, chloroplastic-like	0.34
Metabolite	9,12,13-Trihome	− 0.79
**Glycerophospholipid metabolism**		
Protein	LOC108980768/phospholipase D delta-like	0.46
Metabolite	Choline	− 0.65
**Nicotinate and nicotinamide metabolism**		
Protein	LOC108981116/uridine nucleosidase 1	0.36
Metabolite	Niacinamide	− 0.75
**Porphyrin and chlorophyll metabolism**		
Protein	LOC109000632/pheophorbide a oxygenase, chloroplastic-like	0.27
	LOC109004766/uroporphyrinogen decarboxylase-like	0.30
Metabolite	Hydroxymethylbilane	− 0.77
**Fatty acid biosynthesis**		
Protein	LOC108986581/3-oxoacyl-[acyl-carrier-protein] reductase 4 isoform X1	0.31
	LOC108993714/long chain acyl-CoA synthetase 1-like	0.42
Metabolite	Dodecanoic acid	0.88
	Myristic acid	− 0.84
**Sulfur metabolism**		
Protein	LOC109007939/serine acetyltransferase 1, chloroplastic-like	0.42
Metabolite	Aminoethylsulfonic acid	1.79
	Succinic acid	− 0.88
**Butanoate metabolism**		
Protein	LOC109013191/uncharacterized protein LOC109013191 isoform X3	− 0.30
Metabolite	Hydroxybutanoic acid	0.85
	Butyric acid	− 0.72
	Succinic acid	− 0.88
**Pantothenate and CoA biosynthesis**		
Protein	LOC108982864/ketol-acid reductoisomerase, chloroplastic	0.41
Metabolite	L-Valine	3.04
**Flavonoid biosynthesis**		
Protein	LOC108996947/trans-cinnamate 4-monooxygenase-like	0.34
	LOC108999180/caffeoyl-CoA O-methyltransferase 5	0.71
	LOC108982002/flavonol synthase/flavanone 3-hydroxylase-like	− 0.28
	LOC109010986/shikimate O-hydroxycinnamoyltransferase-like	0.34
Metabolite	Chlorogenic acid	1.19
	Delphinidin	2.52

### Verification of DEP analysis results by PRM

In order to evaluate the validity of the experimental results by iTRAQ analysis, we selected 20 proteins (LOC108981203, LOC108986524, LOC108986997, LOC108987294, LOC108990523, LOC108993800, LOC108995000, LOC108995788, LOC108998475, LOC109003002, LOC109005150, LOC109005909, LOC109007845, LOC109010267, LOC109010467, LOC109010561, LOC109011398, LOC109011575, LOC109012612, LOC109020284) from various pathways, including those of fructose metabolism, glutamine biosynthesis, glucose metabolism, photosynthetic subunit of subcomplex, ribosomal protein, and HSPs, and performed PRM analysis (Table 2, Table S3). The results indicated that 19 proteins (95%) showed similar trends between the iTRAQ analysis and PRM results, indicating the reliability of the iTRAQ analysis, but LOC109005150 analyzed by PRM was not consistent with iTRAQ data.

**Table 2:** PRM verification of DEPs in pecans after 48 h salt stress compared with controls.

No	UniProt accession	Gene*	Protein symbol	Unique peptide	Log_2_ (Fold change)
1	A0A2I4DL15	AT3G09640	LOC108981203	HPDELAHEANNGLDIAVR	− 0.46
2	A0A2I4E5N6	AT4G17030	LOC108986524	TVNDGSVTGVSR	0.15
3	A0A2I4E7J1	AT2G21250	LOC108986997	TVAQIVLR	− 0.56
4	A0A2I4E8K1	AT4G02340	LOC108987294	ALAPDLR	− 0.24
5	A0A2I4EKZ7	AT4G27670	LOC108990523	DGVLYITIPK	4.05
6	A0A2I4EYA1	AT4G10960	LOC108993800	LAGDFGDNLSFHQVDIR	1.54
7	A0A2I4F2X6	AT3G23600	LOC108995000	QFEEVLTAR	− 0.14
8	A0A2I4F5P0	AT1G08550	LOC108995788	IQTPDGGFFTR	− 0.19
9	A0A2I4FFZ4	AT2G29500	LOC108998475	ENSAFANTR	0.34
10	A0A2I4FXX6	AT5G13510	LOC109003002	AEIYAQLLGSLK	0.44
11	A0A2I4G6G4	AT2G36460	LOC109005150	YQDELIANAAYIGTPGK	− 0.17
12	A0A2I4G9H4	AT5G35630	LOC109005909	SILNLSLR	0.09
13	A0A2I4GH76	AT3G16240	LOC109007845	FDDSFSLGSLK	0.13
14	A0A2I4GRR4	AT1G71695	LOC109010267	FVDFMTK	− 0.25
15	A0A2I4GSI9	AT3G06035	LOC109010467	YTGAGIGSEK	− 0.15
16	A0A2I4GSU9	AT1G64770	LOC109010561	YETLDQGR	− 0.25
17	A0A2I4GW66	AT1G12230	LOC109011398	LAYDTHGIIR	− 0.38
18	A0A2I4GWV0	AT2G27290	LOC109011575	VGISTNETGEK	− 0.36
19	A0A2I4H153	AT4G13010	LOC109012612	AVQYNAYGGGPDGLQHVEVPVPTPNKDEVLLR	− 0.15
20	A0A2I4HQ33	AT5G48480	LOC109020284	ASDAIQFYK	0.26

*Gene code was derived from *Arabidopsis thaliana* (TAIR10) (https://www.arabidopsis.org/index.jsp). PRM, parallel reaction monitoring; DEPs, differentially expressed proteins. The related descriptions of these DEPs were shown in Table S1.

## Discussion

Various studies have shown that salt stress has a significant impact on both the photosynthetic structure and efficiency of plant leaves^29,30^. It has been found that the maximum quantum yield of Photosystem II (PSII; Fv/Fm) in salt-sensitive plants is significantly reduced during high salt stress^28^. For example, in the salt-sensitive sweet sorghum, salt stress was also found to significantly reduce *P*n, *C*i, and *G*s^31^. In contrast, the Fv/Fm ratio in salt-tolerant rice was essentially unaffected by salt stress^32^, a finding similar to the results of this study (Fig. 1), and confirming that pecans have a high degree of salt tolerance, the Fv/Fm ratio is one of the iconic indicators of salt-tolerant plants. In addition, it has been reported that down-regulation of expression of the photosystem II 10 kDa protein (PSBR) alleviates light-induced non-photochemical quenching (NPQ) and the decline in Fv/Fm^33^. Since the PSBR plays an important role in maintaining the stability of the PSII reaction center, including repairing damage to the system, to reduce salt-alkali stress. Then, two PSII repair proteins (PSB27-H1 and PSB27) were identified in this study (Table S1), and the small changes observed in *P*n, NPQ, and Fv/Fm may be due to the result of the down-regulation of these proteins. In addition, the data also showed the down-regulation of photosystem I (PSI) reaction center subunits (XI, V, VI, and N) proteins (Table S1). We believe that these may be PSI-protective proteins, with functions similar to that of the PSII repair protein, although this requires verification. Unfortunately, we did not identify DEMs reacting with these photosynthesis-related proteins in the result of the correlation analysis, suggesting that the proteins may play indirect roles in the regulation of the salt-stress response.

As is known, sugar plays a key role in plant stress perception and signal transduction, and can also mediate osmotic regulation and carbohydrate distribution^34^. Consistent with a previous report^35^, this study also identified changes in the levels of various soluble sugars, including sucrose, fructose, and glucose. Twenty DEPs that interacted directly with these sugars were identified, with most of these proteins up-regulated under salt-stress conditions (Fig. 6). This led to the identification of two key metabolites, namely L-fucose and succinic acid. Of these, L-fucose plays an important role in the response of plants to salt stress, and as a defense strategy for protein glycosylation, it participates in regulating the formation of physical barriers (keratin) and activating the immune system, stomata defense, and peptide synthesis^36,37^. Our findings confirm these results. The large increase in L-fucose accumulation (log_2_ Fold change = 2.74) suggests the immune response may be activated under conditions of salt stress. L-fucose, found to be one of the key nodes in the regulatory network of DEPs and DEMs under salt stress, is known to interact with many proteins, including PMM, FKGP, GER2, and APL3, although its precise function is not understood. In contrast, the succinate-interacting protein CSY2, one of the key nodes in the regulatory network, was found to be down-regulated, possibly due to changes in the tricarboxylic acid cycle (TCA cycle) and mitochondrial respiration. CSY2 may thus assist the adaptation to salt stress in the pecan. This is consistent with previous research reports^38^. It is worth noting that both beta-D-fructose 1,6-bisphosphate (log_2_ Fold change = 2.74) and its interaction partner fructose 1,6-bisphosphate aldolase (FBA) 6 were found to be up-regulated (log_2_ Fold change = 0.35). Since the FBA is an important enzyme in the glycolysis/gluconeogenesis pathway, and is located in the chloroplast where it increases carbon flux through the Calvin cycle and assists in stress tolerance by alleviating the inhibition of photosynthesis induced by salt stress^39^. This may be the reason why we did not observe a significant reduction in the photosynthetic rate (*P*n) after 48 h. This is consistent with previous findings^40–42^. We also observed protein enrichment in the phenylpropane synthesis pathway (BGLU12, HCT, and OMT1) together with an unexpected increase in the accumulation of beta-D-Glucosyl-2-coumarinate and *cis*-beta-D-Glucosyl-2-hydroxycinnamate, suggesting that the plants were actively responding to salt stress and repairing oxidative damage. However, the chlorogenate showed down-regulation (Table 1), it is clearly the result of salt stress, the precise function requires clarification. Similarly, this study also found that proteins and metabolites involved in the biosynthetic pathway of flavonoids were also enriched (Figs. 2B, 5), including the up-regulated C4H, HCT, CCoAOMT1, chlorogenic acid, and delphinium, consistent with previous reports^43–45^. This indicates that the flavonoid biosynthetic pathway in plants is positively regulated under salt stress. In addition, the secondary metabolites of polyphenols, chlorogenic acid and delphinium are known antioxidants and may participate in the response to the adverse effects of salt stress through plant osmotic adjustment. Finally, the protein and metabolite regulatory networks appear to be independent, and the α-linolenic acid and linoleic acid metabolic pathways were not found to be related to other metabolic pathways (Fig. 6). Among them, 12-OPDA is an oxidation product of α-linolenic acid, and as a precursor of jasmonic acid that is an important plant hormone. It has an important regulatory role in the plant stress response^46,47^, and will serve as a key research topic in the future.

Briefly, most of the current research results on the molecular mechanism of salt stress have been undertaken in model plants and food crops, and this is the first comprehensive study to incorporate plant physiology, proteins, and metabolites in an investigation of the responses to salt stress in pecan. The study also provides a theoretical basis for the choice of suitable economically viable trees in high-salt tidal areas. Further functional analysis and genetic translational verification of the key proteins identified is, however, still needed.

## Conclusion

This study analyzed the photosynthetic and fluorescence parameters of pecans under salt stress using iTRAQ and untargeted LC/MS to investigate the response of pecans to salt stress. The results showed the differential expression of 198 DEPs and 538 DEMs after 48 h of salt stress. Further investigation using protein and metabolomics pathway correlation analysis showed enrichment of 21 core pathways and two key nodes of the salt stress regulatory network, L-fucose and succinate. Finally, PRM analysis verified the expression levels of multiple proteins. In summary, this study provides an important theoretical starting point for future coastal saline-alkali cultivation and related research.

## Supplementary Information

Supplementary Information.

Supplementary Figure S1.

Supplementary Table S1.

Supplementary Table S2.

Supplementary Table S3.
