# Supplementary material for: Integrated physiological, proteomic, and metabolomic analyses of pecan cultivar ‘Pawnee’ adaptation to salt stress
Source: Sci Rep. 2022 Feb 3;12:1841. doi: 10.1038/s41598-022-05866-9 (PMC8814186; doi:10.1038/s41598-022-05866-9)
Supplement: Supplementary file 1 — Supplementary Information. [file 41598_2022_5866_MOESM1_ESM.doc]

**Supplementary Materials:**

**Figure legends**

### Figure S1. Morphological consequences in the pecan leaf after 0, 24, and 48 h of salt treatment (0.6 % NaCl).

**Tables**

**Table S1.** Quantification of the differentially expressed proteins in pecan under salt stress (0.6 % NaCl, 48 h) by iTRAQ.

**Table S2.** Quantification of the differentially expressed metabolites in pecan under salt stress (0.6 % NaCl, 48 h) by LC/MS non-targeted metabolomics technology.

**Table S3.** Analysis of candidate peptide fragments of target proteins in pecan under salt stress (0.6 % NaCl, 48 h) compared with controls.
