# Supplementary figures and images for: Integrated physiological, proteomic, and metabolomic analyses of pecan cultivar ‘Pawnee’ adaptation to salt stress
Source: Sci Rep. 2022 Feb 3;12:1841. doi: 10.1038/s41598-022-05866-9 (PMC8814186; doi:10.1038/s41598-022-05866-9)

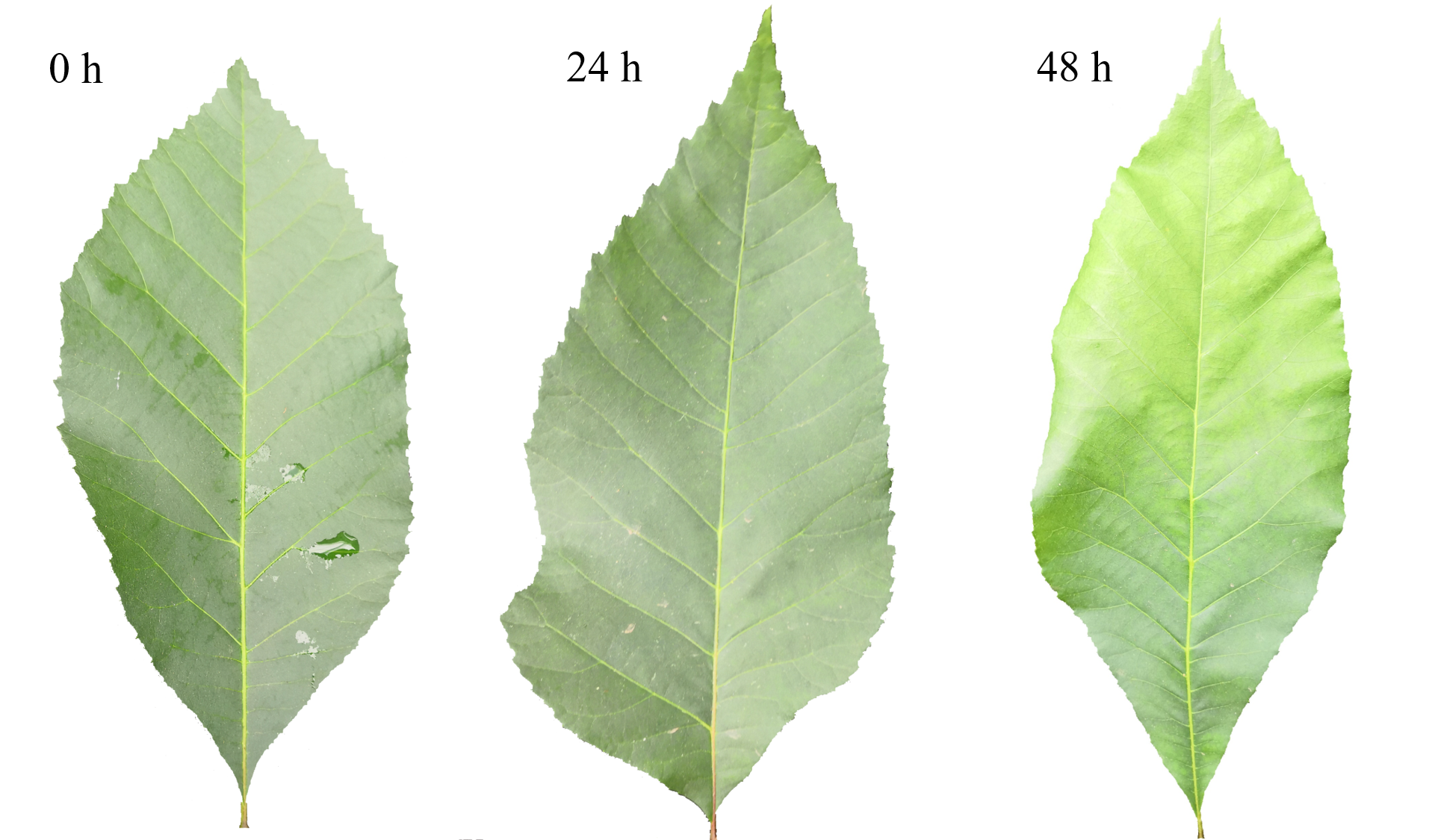

Supplement: Supplementary file 2 — Supplementary Figure S1. [file 41598_2022_5866_MOESM2_ESM.tif]
